# Case Report: Successful treatment of advanced hepatocarcinoma with the PD-1 inhibitor Camrelizumab

**DOI:** 10.3389/fimmu.2023.1221418

**Published:** 2023-07-27

**Authors:** Wenling Ye, Lihong Cai, Minjie Zhang, Yali Wu, Huina Sun, Yan-Dong Wang, Yubing Xia

**Affiliations:** ^1^ Key Laboratory of Receptors-Mediated Gene Regulation, School of Medicine, Henan University, Kaifeng, China; ^2^ Kaifeng Key Laboratory of Radiation Oncology, Kaifeng Cancer Hospital, Kaifeng, China; ^3^ State Key Laboratory of Chemical Resource Engineering, College of Life Science and Technology, Beijing University of Chemical Technology, Beijing, China

**Keywords:** primary liver cancer, immune checkpoint inhibitor, PD-1, Camrelizumab, case report

## Abstract

Primary liver cancer is characterized by closely related with chronic liver inflammation, thereby reversing hypoxic immunosuppressive microenvironment of tumor cell growth by immunotherapy drug is a potentially effective strategy. Camrelizumab is an anti-PD-1 antibody being developed by Jiangsu Hengrui Pharmaceuticals Co., Ltd. We reported a case of an adult critical Chinese patient with primary hepatocellular carcinoma and lung metastasis completely responding to Camrelizumab, most of the lesions were stable and no new lesions occurred after 1-year treatment, which provides us to reconsider the therapeutic effect of Camrelizumab on such patients. Camrelizumab had a safety profile for the patient in our case report, except for the occurrence of RCCEP. This case provides the evidence of the effective antitumor activity and manageable toxicities of Camrelizumab for patients with advanced hepatocellular carcinoma, which was the first application as far as we know.

## Introduction

Primary liver cancer represents the sixth most dominant cancer worldwide and making it the third leading cause of cancer death globally (1). Surgery, liver transplantation, local ablative therapies, radiotherapy and chemotherapy are the most popular traditional therapies. Currently, immune checkpoint blockade is an advanced strategy, and has rapidly evolved into the most popular form of cancer immunotherapy with remarkable clinical benefits (2). Among the emerging checkpoint inhibitors, including PD-1/PD-L1, CTLA-4, LAG3, TIM3 and VISTA (3), PD-1/PD-L1 is one of the most promising targets in immunotherapy (4). To date, 9 FDA-approved therapeutic antibodies targeting PD-1/PD-L1 have achieved significant clinical effects and long-term remission in the indications (5).

Camrelizumab is an anti-PD-1 antibody being developed by Jiangsu Hengrui Pharmaceuticals Co., Ltd. for the treatment of relapsed or refractory classic Hodgkin’s lymphoma, hepatocellular carcinoma and other malignant tumor types (6). We herein present a patient with hepatocellular carcinoma who was treated with camrelizumab.

## Case presentation

**Figure 1:**
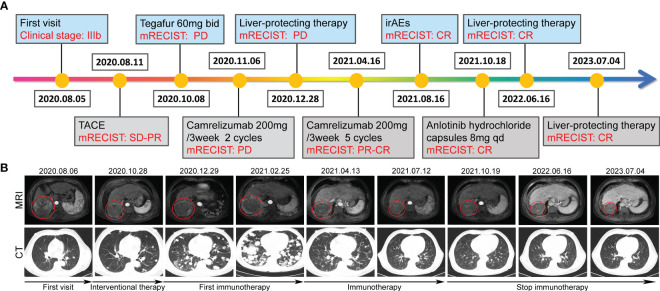
Tumor responses after therapy. **(A)** Timeline of disease status and corresponding treatment regimens. **(B)** Time line of treatments of the patient and changes in MRI and CT of the liver and lung during treatments, the red circle location of the tumors.

**Figure 2:**
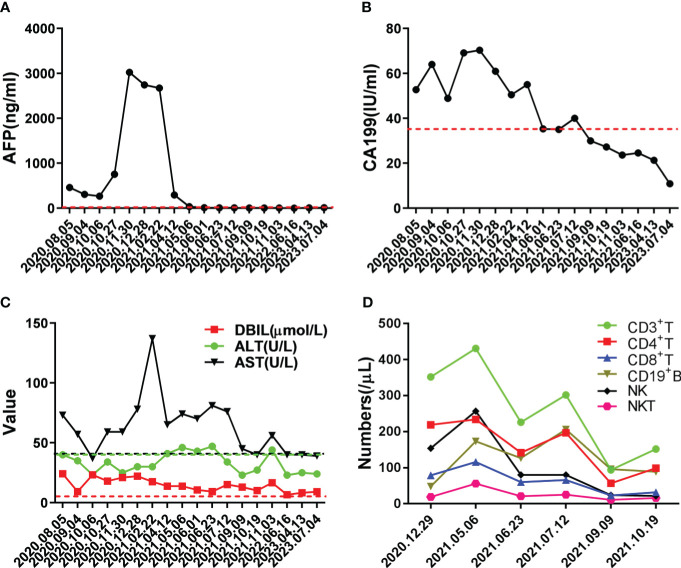
Changes in important indicators during the course of the disease. **(A)** Changes in level of tumor marker AFP during treatment, the red dotted line indicated normal level. **(B)** Changes in level of tumor marker CA199 during treatment, the red dotted line indicated normal level. **(C)** Changes in levels of liver function during treatment, the dotted lines indicated normal level. **(D)** Changes in numbers of immune cells during treatment; medical reference range: CD3^+^ T, 723-2737/μL; CD4^+^ T, 404-1612/μL; CD8^+^ T, 220-1129/μL; CD19^+^ B, 80-616/μL; NK, 84-724/μL; NKT, 40-300/μL.

In August 2020, an adult Chinese patient with no history of disease presented with a intermittent abdominal distension/discomfort, and Eastern Cooperative Oncology Group (ECOG) performance status score of 1. CT scan showed occupying lesion in the right lobe of the liver, and multiple small nodules in both lungs. Combined with an extremely high serum alpha-fetoprotein (AFP) level (461.48 ng/ml), the patient was diagnosed with primary hepatocellular carcinoma and lung metastasis (stage IIIb, CNLC stage, Child-Pugh A with 6 points) (**Figures 1**, **2**). The patient had a half-year history of chronic hepatitis B with oral antiviral therapy (entecavir dispersible tablets, 0.25g qd).

On August 11, 2020, the patient received interventional therapy for liver cancer performed under DSA-guided local anesthesia, using gelatin sponge embolization, iodized oil, drug-carrying microspheres, lobaplatin injection 20mg, fluorouracil injection 0.5g, chemoembolization of 100 mg epirubicin dose. After interventional therapy for the patient, stable disease-partial response (SD-PR) was evaluated according to mRECIST solid tumor response evaluation criteria. On September 8, 2020, the patient underwent the second interventional therapy. Subsequently, he accepted Gimeracil and Oteracil Potassium Capsules (Tegafur, 60mg bid) chemotherapy on October 8, 2020. The patient came back to the hospital on October 27, 2020, due to the aggravation of abdominal distention. Chest CT scan indicated a significant increase in lung metastases.

On November 6, 2020, the patient accepted one course of Camrelizumab treatment with 200 mg once daily in 21-day cycles. Afterward, the patient presented with the prodromal symptoms of hepatic encephalopathy, including irritability, irrelevant answer, lethargy, and disturbance of consciousness. Then deamination treatment with arginine did improve this critically severe patient. Subsequently, the patient underwent second round of Camrelizumab treatment on December 4, 2020. Later, he refused to continue immunotherapy due to economic reasons. The patient was given best supportive care using oral antiviral entecavir tablets (0.5mg qd) and glycyrrhizin tablets (75mg tid). On February 25, 2021, on MRI of the abdomen, dense masses were seen in the right posterior lobe of the liver, multiple nodules and increased lesions were observed in the liver. Multiple enlarged high-density nodules were observed in both lungs, significantly increased in number and volume.

**Figure 3:**
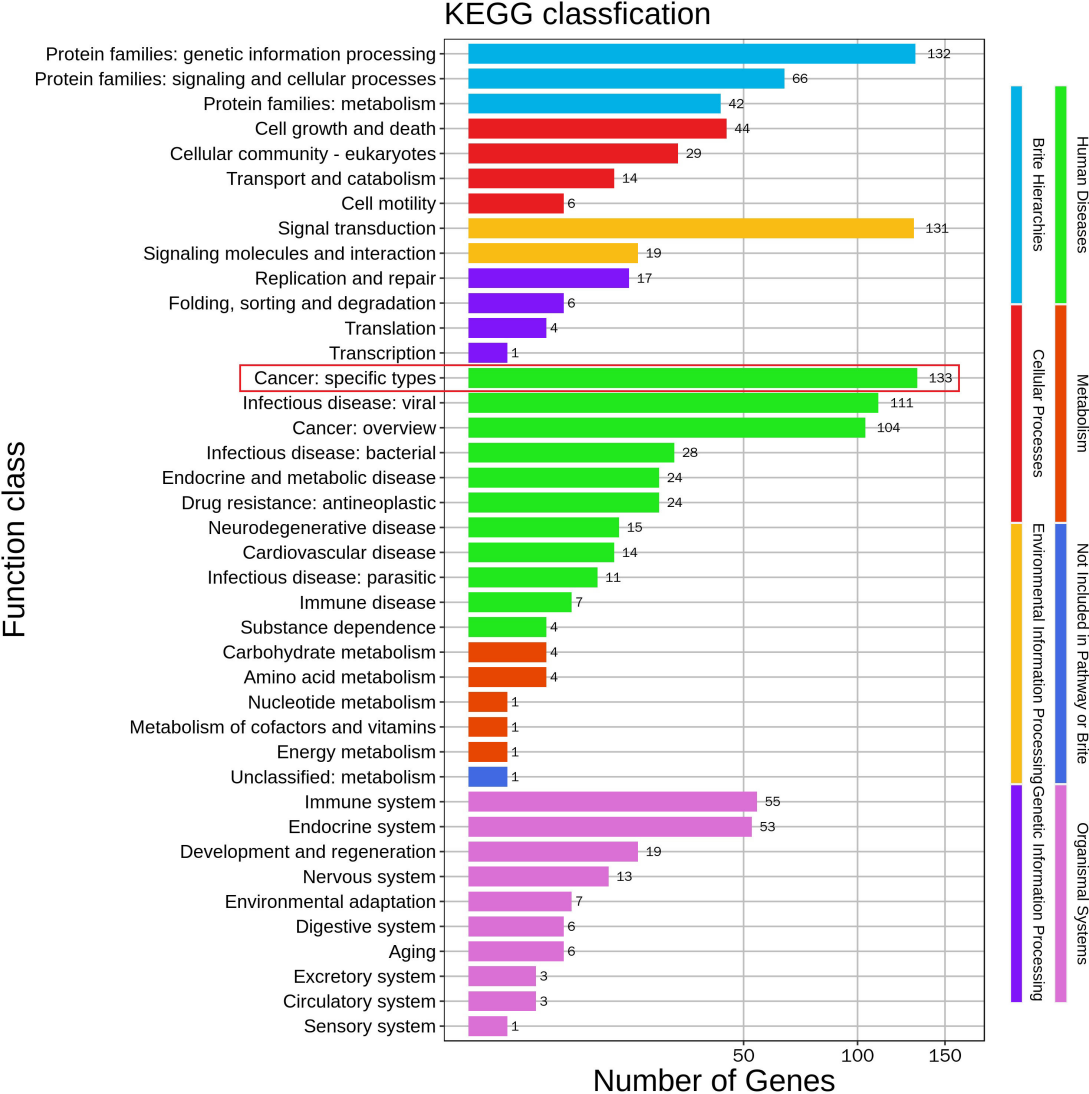
Significantly enriched KEGG pathway terms by analyzing mutated genes in the patient.

Life is full of surprises. On April 12, 2021, CT imaging and MRI revealed a low-density mass in the right posterior lobe of the liver, and a slightly enhancement in nidus during the arterial phase, portal phase, and delayed phase. Compared with the status on February 25, 2021, the number and volume of lesions was decreased significantly in both liver and lung. Subsequently, the patient underwent another five rounds of Camrelizumab treatment. CT imaging and MRI revealed sustained reductions for the number and volume of lesions in the liver and lung. Meanwhile, tumor markers AFP and CA199, as well as liver function indexes, returned to normal level (**Figure 2A-C**). At present, few reports on peripheral immune cells in the early stage of immunotherapy have been found. Some studies have confirmed that the initial stage of immunotherapy showed the increased activity of peripheral blood CD8^+^ and CD4^+^ T cells (7, 8). In this case, the immune cells of patients, such as T cells, B cells, NK cells, and NKT cells, were lower than normal values. But on May 6, 2021, the number of immune cells was significantly increased which coincided with the decrease in the size of nodules and lesions, after homeostasis in immunosurvilance, that the count of all cells are partially reduced. This may clarify the role of humoral immunity in response to cancer cells (**Figure 2D**). DNA sequencing of the patient’s blood cells in Jun 2021 revealed that the patient did harbor a certain extent gene mutation burden, including metabolism, cell growth and death, hepatocellular carcinoma, and immune-related mutations (9, 10). Moreover, 8 mutated genes (*APC*, *AXIN2*, *CDKN1A*, *EGFR*, *PIK3CA*, *PTEN*, *TCF7L2* and *TP53*) significantly relating to the hepatocellular carcinoma pathway were identified (11–15) (**Figure 3**).

**Figure 4:**
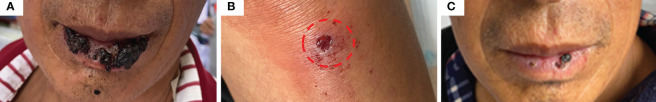
The patient developed grade 2 RCCEP after Camrelizumab treatment. **(A, B)** the patient appeared grade 2 RCCEP; **(C)** RCCEP was improved after drug withdrawal.

The patient appeared reactive cutaneous capillary endothelial proliferation (RCCEP, grade 2) in August 2021, which was improved after drug withdrawal (**Figure 4**). Afterward, the patient accepted oral androtinib hydrochloride capsules with 8 mg qd d1-14 in 21-day cycles. Fortunately, lesions in the liver and lung of the patient showed significant complete response (CR), and the ECOG performance status score was 1. Since January 2022, the patient has not received antitumor therapy, and symptomatic supportive care using oral antiviral entecavir tablets (0.5mg qd) and glycyrrhizin tablets (75mg tid), the imaging evaluation was CR. By December 2022, the patient was stable during telephone follow-up. When the patient was reexamined in July 2023, the imaging evaluation was CR and the ECOG performance status score was 0.

## Discussion

Immune escape is one of the hallmarks of cancer that tumor cells can frequently evade immune surveillance through overexpressing PD-L1. Anti-PD-(L)1 monoclonal antibodies has opened new avenues in treating several types of cancer. As HCC carcinogenesis and progression are closely related with liver chronic inflammation, reversing chronic inflammation-dependent immunosuppressive microenvironment of HCC is an effective strategy. Clinical trials assessing the effects of immune checkpoint inhibitors (ICI) in HCC are currently in progress (16, 17). Despite the fact that ICIs have potential and suppressing effects against liver cancer, only a small number of patients significantly responded to the treatment. Due to the strategic position and unique function, the liver predominantly tended to tolerance rather than existing in a reactive state, which may make the immunotherapy even more challenging against HCC (18).

In this case report, a primary hepatocellular carcinoma and lung metastasis patient responded well to Camrelizumab immunotherapy, most of the lesions were stable and no new lesions occurred after 1-year treatment, which provides us to reconsider the therapeutic effect of Camrelizumab on such patients. There is evidence that anti-PD-(L)1 immunotherapy after chemotherapy may play an important role in the tumor immune microenvironment and decrease hyperprogressive disease (19). The patient in our case report, accepted Camrelizumab immunotherapy after chemotherapy, and showed significant CR. While the precise mechanism of drug action is a mystery, and specific factor responsible for tumor regression remains unclear. Owing to the effective response of this case, we will continue to pay attention to the patient’s subsequent disease progresses. Moreover, since the tissue samples of the patient before and after treatment were insufficient to detect biomarkers of the tumor microenvironment, it was difficult to confirm whether chemotherapy before immunotherapy achieved a synergistic effect with Camrelizumab. Confusingly, after two Camrelizumab treatments on November 6, 2020 and December 4, 2020, the tumors experienced significant progression according to the image evaluation results on February 25, 2021, whereas the lesions both in liver and lung regressed significantly on April 12, 2021. The retrospective analysis revealed the images from February 25, 2021 might show pseudoprogression, and the immunotherapy actually remained in effect (20). Therefore, we observed the regression of liver and lung lesions on April 12, 2021. Fortunately, our assessment suggests that this patient benefits from the sustained immune response. Overall, complete regression of advanced hepatocellular carcinoma following Camrelizumab immunotherapy with manageable toxicities is a relatively rare but encouraging event. Additional research is needed to elucidate the possible mechanisms of drug action, which will provide a new promising first-line option for patients with advanced hepatocellular carcinoma.

## Data availability statement

The datasets presented in this study can be found in online repositories. The names of the repository/repositories and accession number(s) can be found below: PRJNA980414 (SRA).

## Ethics statement

The studies involving human participants were reviewed and approved by Kaifeng Cancer Hospital Reviewer Board. The patients/participants provided their written informed consent to participate in this study. Written informed consent was obtained from the participant/patient(s) for the publication of this case report.

## Author contributions

WY, LC performed the experiments, analyzed the data and wrote the manuscript. MZ, YW, HS contributed to the collection of data. YX, Y-DW contributed to the conception, design, revision and final approval of the submitted version. All the authors contributed to the article and approved the submitted version.
